# The Effects of Acute Waterborne Exposure to Sublethal Concentrations of Molybdenum on the Stress Response in Rainbow Trout, *Oncorhynchus mykiss*

**DOI:** 10.1371/journal.pone.0115334

**Published:** 2015-01-28

**Authors:** Chelsea D. Ricketts, William R. Bates, Scott D. Reid

**Affiliations:** Department of Biology, Irving K. Barber School of Arts and Sciences, University of British Columbia, Okanagan campus, 3333 University Way, Kelowna, British Columbia, Canada, V1V 1V7; CINVESTAV-IPN, MEXICO

## Abstract

To determine if molybdenum (Mo) is a chemical stressor, fingerling and juvenile rainbow trout (*Oncorhynchus mykiss*) were exposed to waterborne sodium molybdate (0, 2, 20, or 1,000 mg l^-1^ of Mo) and components of the physiological (plasma cortisol, blood glucose, and hematocrit) and cellular (heat shock protein [hsp] 72, hsp73, and hsp90 in the liver, gills, heart, and erythrocytes and metallothionein [MT] in the liver and gills) stress responses were measured prior to initiation of exposure and at 8, 24, and 96 h. During the acute exposure, plasma cortisol, blood glucose, and hematocrit levels remained unchanged in all treatments. Heat shock protein 72 was not induced as a result of exposure and there were no detectable changes in total hsp70 (72 and 73), hsp90, and MT levels in any of the tissues relative to controls. Both fingerling and juvenile fish responded with similar lack of apparent sensitivity to Mo exposure. These experiments demonstrate that exposure to waterborne Mo of up to 1,000 mg l^-1^ did not activate a physiological or cellular stress response in fish. Information from this study suggests that Mo water quality guidelines for the protection of aquatic life are highly protective of freshwater fish, namely rainbow trout.

## Introduction

Molybdenum (Mo), as molybdate (MoO_4_^2-^), is a common component of natural freshwaters due to erosion and weathering of ores from igneous and sedimentary rock, especially shale [1,2]. In unimpacted areas freshwater levels of Mo rarely exceed 0.02 mg l^-1^ [1]. Anthropogenic loading of Mo into aquatic and terrestrial ecosystems occurs by Mo mining, milling, and smelting, combustion of fossil fuels, uranium and copper mining and milling, oil refining, shale oil production, and Mo containing fertilizers [1, 3–8]. Elevated levels of Mo such as 1.4 mg l^-1^ [9], 11.4 mg l^-1^ [10], 32.5 mg l^-1^, [11], and 100 mg l^-1^ [12] have been reported in freshwaters receiving mining discharge. There is potential for additional release and distribution of Mo into the aquatic environment as world production of this valued natural resource, which is primarily used as an alloying agent, continues to increase [13, 14].

Molybdenum is an essential micronutrient and forms the catalytic center of over 50 enzymes including xanthine oxidase, sulphite oxidase, aldehyde oxidase and DMSO reductase [15–17]. In domestic animals, deficiency symptoms include depressed growth, impaired reproduction, poor fetal survival, and decreased molybdoenzyme activities [18, 19]. Deficiency signs and requirements of Mo remain to be established in fish [20].

Little is known about the effects of elevated Mo levels on living systems, especially fish. Most of the available information is based on gross morphological changes or readily visible symptoms in domestic animals [1,4]. Toxicity tests in freshwater fish have reported 96 h LC_50_ Mo (added as sodium molybdate) concentrations ranging from >50 to >10,000 mg l^-1^ [9, 21–26]. Manifestations that have been reported as a result of Mo exposure include fused gill lamellae, gut and pyloric caeca hemorrhaging, pale livers with hemorrhaging along liver margins, pale kidneys [22], inhibition of spermatogenesis, decreased gonadosomatic index [27], darker appearance, increased mucus production, higher ventilatory frequencies, post-exercise loss of equilibrium, and exercised-induced delayed mortality [26]. According to these studies, it appears that Mo, at high concentrations, does have physiological effects and can result in death. Therefore, more studies regarding the effect of Mo on other systems, such as the stress response, need to be investigated.

The stress response enables fish to avoid or cope with environmental, physical, or biological changes and thus maintain internal homeostasis. Fish, like other vertebrates, exhibit both a physiological and a cellular stress response. The physiological stress response consists of primary endocrine responses (secretion of cortisol and catecholamines), secondary metabolic responses, including an increase in glucose and hematocrit, and tertiary or whole organisms responses [28]. Elevated cortisol and glucose levels have been reported for fish exposed to a variety of physical [29, 30] and chemical stressors [31–33], including metals [34–36].

The cellular stress response is facilitated by the action of various stress proteins such as the heat shock protein (hsp) and the metallothionein (MT) families. Heat shock proteins are molecular chaperones that function by regulating cellular homeostasis through ensuring proper folding, transport, and degradation of proteins. The two main hsp families are hsp70, consisting of the constitutively expressed hsp73 and the stress inducible hsp72, and hsp90. In fish, these proteins are induced in cell lines, primary cell cultures, and whole organisms by a variety of stressors including industrial effluents [37–39], pesticides [40, 41], pathogens [42, 43], and metals [44–49]. Exposure to stress causes proteins to denature, misfold, or unfold, ultimately exposing hydrophobic regions and causing protein aggregation. The heat shock response is therefore essential to maintain proper protein structure and cellular function. Metallothionein is a metal binding protein with a high affinity for groups Ib and IIb transition metals [50]. One of the suggested functions of MT is to regulate essential trace metals like zinc and copper and detoxify metals such as cadmium and copper. Synthesis of MT is induced to a greatest degree by exposure to metals and to a lesser degree by hormones, cytokines, and organic contaminants [51].

The objectives of the present study were to determine the effect of sublethal concentrations of waterborne Mo on (1) the physiological stress response, as measured by plasma cortisol, blood glucose, and hematocrit, and (2) the cellular stress response, as measured by total hsp70 (72 and 73), hsp72, hsp90, and MT induction in rainbow trout, a species that has proven to be a very useful model system for understanding the effects of many other metals [52].

## Materials and Methods

### Animals

This study was approved by the University of British Columbia Animal Care Committee (AUP A10–0301) and conforms to guidelines outlined by the Canadian Council on Animal Care. Juvenile (mass range 215–509 g) and fingerling (mass range 2–18 g) rainbow trout, *Oncorhynchus mykiss*, were obtained from Campbell Lake Trout Hatchery (Little Fort, BC, Canada; Mo ≤ 1.0 μg l^-1^) and transferred to the University of British Columbia Okanagan (Kelowna, BC, Canada). Fish were kept indoors under a 12 h light/dark photoperiod in polyethylene tanks: 600 l for juveniles and 70 l for fingerlings. Tanks received a constant supply of aerated, dechlorinated City of Kelowna (Glenmore-Ellison Improvement District; Mo ≤ 1.0 μg l^-1^) tap water at a temperature range of 10 to 13°C. Trout were acclimated and held under these conditions for 3 months prior to experimentation. The fish were fed 2% body weight every other day with a commercial feed (Aqua Pride Trout Pellets; Hi-Pro Feeds LP), but received no food for 48 h prior to and throughout all experiments.

### Cannulation procedure

Juvenile rainbow trout were anesthetized with 0.1 g l^-1^ MS-222 (ethyl *m*-aminobenzoate methanesulfonate) adjusted to pH 7.5 with NaHCO_3_ and then placed onto an operating table to allow continuous retrograde irrigation of the gills with anesthetic solution. An indwelling cannula (Clay-Adams PE 50 tubing) was implanted into the dorsal aorta according to standard techniques [53]. Fish were revived on the operating table by irrigation of the gills with aerated water and then placed into individual chambers (see section 2.3). Fish were allowed to recover from the effects of anesthesia and surgery for 48 h before experimentation commenced. Cannulas were flushed daily with 0.2–0.3 ml of heparinized (100 i.u. ml^-1^ ammonium heparin) Cortland saline to prevent clotting [54].

### Exposure system

Forty-eight hours prior to Mo exposure fish were randomly transferred to black plexiglass chambers (3.0–3.5 l; 1 juvenile/chamber or 8 fingerlings/chamber) in order to recover from surgery and to acclimate to the experimental system. Each chamber was supplied with a flow of water and constant aeration. All chambers were half-submerged on a continuously flowing wet table to maintain water temperature during the static exposure. At time 0, flow to the chambers was stopped and water was replaced with the desired Mo concentration. In doing so, one-third of the chamber was allowed to drain, fish were always fully submerged, and then the chamber was flushed with 18 L of solution before being refilled. Fish were exposed to waterborne sodium molybdate dihydrate (Na_2_MoO_4_•2H_2_O; Fisher Scientific, Pittsburgh, PA, USA) for 96 h at 0 (existing Mo in the water was ≤ 0.0010 mg l^-1^), 2, 20, or 1000 mg l^-1^ of Mo. The lowest exposure concentration of 2 mg l^-1^ was chosen as it is the water quality limit for the protection of freshwater aquatic life proposed by Swain [55] and adopted by the province of British Columbia. When included, the highest exposure concentration of 1000 mg l^-1^ was chosen as it was 50% of the 96 h LC_50_ estimate for freshwater fish native to Okanagan water (Reid 2002). Furthermore, chosen exposure concentrations overlap with molybdenum concentrations that we have previously shown to accumulate internally, gill and liver, in freshwater fish held and exposed under nearly identical conditions (0–250 mg l^-1^, [26]). To ensure that water quality and Mo levels were adequately maintained water was changed, as previously described, every 8 h. Temperature (10–13°C), general hardness (140 ± 12 mg l^-1^ CaCO_3_), pH (8.0 ± 0.2), and ammonia (0.92 ± 0.11 mg l^-1^) were constantly monitored.

### Treatments and sampling

**Juvenile fish.** Non-cannulated (318.3 ± 20.9 g, N = 18) juvenile fish were exposed to 0, 2, or 20 mg l^-1^ of Mo. The experiment was conducted three times and each experiment used two fish per treatment. At 96 h, fish were quickly captured with minimal disturbance and stunned by a cephalic blow. Blood (0.5 ml) was withdrawn by caudal puncture using a syringe previously rinsed with heparinized Cortland saline. Immediately upon collection, whole blood was centrifuged at 10,000 rpm for 2 min to separate the plasma from the erythrocytes. Both plasma and erythrocyte samples were stored at -80°C. Following blood collection, the gills were perfused with Cortland saline and excised from both the fish and the gill arch. The liver was removed and the heart excised without the bulbous arteriosus. All tissues were flash frozen in liquid nitrogen and stored at -80°C until analysis.

**Cannulated juvenile fish.** Cannulated juvenile fish (342.1 ± 16.9 g, N = 18) were exposed to 0, 2, or 20 mg l^-1^ of Mo; six fish per treatment. Fish had their dorsal aortic cannulas sampled prior to initiation of exposure (0 h) and at 8, 24, and 96 h during exposure. Blood (0.5 ml) was sampled using a syringe previously rinsed with heparinized Cortland saline and processed as previously described. Plasma samples were stored at -80°C until analysis. To partially maintain blood volume, approximately 0.2–0.3 ml of Cortland saline was reinjected into the cannula after each blood collection.

**Fingerling fish.** Fingerling fish (11.1 ± 0.9 g, N = 96) were exposed to 0, 2, 20, or 1,000 mg l^-1^ of Mo. The experiment was done three times at 16 fish per treatment. Fish were sampled with minimal disturbance from each chamber prior to initiation of exposure (0 h) and at 8, 24, and 96 h during exposure. Fish were sacrificed from a cephalic blow. Less than 0.1 ml of blood was collected into a heparinized hematocrit tube by means of capillary action after severing the caudal peduncle. The gills, excluding the arch, and liver were excised, flash frozen in liquid nitrogen, and stored at -80°C until analysis.

### Determination of tissue molybdenum content: ICP-MS

To verify that our molybdenum exposures resulted in significant accumulation tissue molybdenum in trout we measured plasma, gill, white muscle and liver molybdenum content from juvenile fish using ICP-MS; sample quantities were insufficient to do so with fingerlings. Tissues samples for ICP-MS analysis were weighed and placed in constant temperature drying oven until a constant dry weight was obtained; approximately one week. A 50 mg aliquot of dry tissue was placed in an acid-washed 15 ml Teflon test tube. 2 ml of concentrated trace metal free nitric acid was pipetted into each tube and placed in a 75°C water bath for 24 h. Tubes were allowed to cool to room temperature then 0.5 ml of peroxide was added and the samples placed back a 46 °C water bath for 18 h to complete sample oxidation. The lower temperature was required for this portion of the digestion to avoid sample loss due to over-active bubbling. The temperature was then increased to 80 °C for an additional 24 h. Once digestion was complete, samples were removed from the bath and placed in a rack beneath an air manifold for drying. Once dry, samples were reconstituted in 10 ml of 1% trace metal free nitric acid and left to settle overnight. 100 μl aliquots of digests were drawn off and placed into a fresh, acid-washed 15 ml Falcon tube and topped off to 10 ml with 1% trace metal free nitric acid. Each sample then received 100 μl of Yttrium (1 ppm stock solution, diluted from 1000 ppm standard) for ICP-MS analysis. Yttrium was added to all samples as an internal standard and to correct for any potential matrix discrepancies. A standard curve was prepared in the same concentrated trace metal free nitric acid matrix (0, 1.0, 10.0 and 100.0 ppb; μg l^-1^). These standards also contained 10 ppb (μg l^-1^) Yttrium as an internal standard. The standard curve was used to convert the output of the ThermoFisher Element XR double-focusing ICP-MS from counts per second to a concentration in parts per billion (ppb). We measure the molybdenum isotope Mo^97^ because it was one of two isotopes (Mo^95^, Mo^97^) that did not have any possibility for interference from other elements that were in our samples and it yielded the most consistent results.

### Determination of hematocrit, blood glucose, and plasma cortisol

Hematocrit levels were measured in duplicate by means of micro hematocrit tubes. Tubes were spun at 2,510 rpm for 5 min on a clinical centrifuge (International Equipment Company, Needham, MA, USA) fitted with a hematocrit head. Blood glucose levels were measured in duplicate using a Precision Xtra glucose meter (Abbott Laboratories, Abbott Park, IL, USA). Total plasma cortisol levels were determined in duplicate using an enzyme-linked immunosorbent assay kit (Neogen Corporation, Lexington, KY, USA), read at 630 nm on an OpsysMR microplate reader (Dynex Technologies, Chantilly, VA, USA).

### Protein extraction and quantification

Tissues and erythrocytes were sonicated in ice-cold lysis buffer (1:1 w/v) containing 100 mM Tris-HCl pH 7.5, 0.1% SDS (sodium dodecyl sulfate), and a SigmaFAST protease inhibitor tablet (Sigma-Aldrich, St. Louis, MO, USA). The lysates were cleared in a microcentrifuge at 10,000 g for 3 min at room temperature. A 10 μl aliquot of supernatant was taken for protein determination with the bicinchoninic acid (BCA) assay using bovine serum albumin (BSA) as a standard. Standards and samples were read at 490 nm. The remaining supernatant was mixed 1:1 with SDS-sample dilution buffer [56] and boiled for 3 min. In order to equalize total protein concentration samples were further diluted in 1X SDS-sample dilution buffer before being frozen at -80°C.

### Hsp70, hsp90, and MT analysis

Protein levels were measured using the discontinuous SDS-polyacrylamide gel electrophoresis (PAGE) method of Laemmli [56]. For hsp analysis, total protein (10 μg) from heart, gill, liver, and erythrocyte samples was resolved with a 4% stacking gel and a 12% separating gel. For MT analysis, 25 μg of liver and 27.5 μg of gill tissue were resolved with a 4% stacking gel and a 15% separating gel. Samples were run alongside Precision Plus Protein dual-color standards (Bio-Rad, Hercules, CA, USA). For hsp72 analysis, a liver sample from the same heat-shocked fish was loaded, in at least duplicate, onto each gel to allow direct comparison among gels. A purified bovine brain standard (H-9776; Sigma-Aldrich) and a purified human hsp90 standard (SPP-770; Assay Designs, Ann Arbor, MI, USA), and a liver sample from the same control fish were used for total hsp70 (72 and 73), hsp90, and MT analysis, respectively. Gels were run in a Mini-Protein II electrophoretic cell (Bio-Rad) containing 1X running buffer at 75 V until samples reached the separating gel at which time the voltage was increased to 150 V for approximately 1 h. Gels were then transferred to Hybond-P PVDF membrane (GE Healthcare, Little Chalfont, Buckinghamshier, UK) in a Mini Trans-Blot electrophoretic transfer cell (Bio-Rad) for 1 h at 100 V for hsp analysis or 80 V for MT analysis. After transfer, the membrane was stained with Ponceau-S stain [0.5% (w/v) Ponceau-S red and 1% (v/v) acetic acid] for 5 min to determine the success of the transfer and equal loading. Membranes containing erythrocyte protein did not stain well with Ponceau-S; therefore, equal protein loading was detected by immunoblotting using a monoclonal mouse anti-GAPDH (glyceraldehyde 3-phosphate dehydrogenase) primary antibody (CSA-335; Assay Designs).

Membranes were blocked with 5% skim milk powder in TBS-T (20 mM Tris, 500 mM NaCl, and 0.1% Tween 20; pH 7.6) for 1 h. Membranes were washed in TBS-T and then incubated for 1 h in primary antibodies diluted in TBS-T. Rabbit anti-salmonid hsp72 (AS05061; Agrisera, Vännäs, Sweden), mouse anti-bovine total hsp70 (72 and 73) (H-5147; Sigma-Aldrich), or rabbit anti-salmonid hsp90 (AS05063; Agrisera) were all used at 1:5,000 for liver, heart, and erythrocyte samples and at 1:20,000, 1:5,000, and 1:20,000, respectively, for gill samples. The rabbit anti-cod (KH-1) MT primary antibody (M04406210–500; Biosense Laboratories, Bergen, Norway) was used at 1:1,000 for liver samples and 1:500 for gill samples. Membranes were then washed twice for 10 min each in TBS-T and, for hsp72, incubated for 1 h in secondary antibody (SAB-100 goat anti-mouse IgG HRP conjugate; Assay Designs) at a 1:10,000 dilution in TBS-T. For total hsp70 and hsp90 detection, membranes were incubated for 1 hr in goat anti-rabbit IgG HRP conjugate (SAB-300; Assay Designs) at a 1:1000 dilution in liver, heart, and erythrocyte samples and 1:40,000 dilution in gill samples. The same goat anti-rabbit antibody was used for MT detection at 1:5,000 for liver samples and 1:6,000 in gill samples. After this final incubation, membranes were washed 3 times for 10 min in TBS-T. All incubations and washes were done on an orbital shaker.

### Densitometry

Proteins were detected by enhanced chemiluminescence using the ECL Western Blotting Analysis System (GE Healthcare) and Hyperfilm (GE Healthcare). The autoradiography film was developed using KODAK GBX Developer and Replenisher and Fixer and Replenisher (Sigma-Aldrich). Several different exposure times were taken for each blot to ensure linearity of band densities. Bands were scanned using an AGFA SnapScan e50 scanner and quantified using Quantity One software (Version 4.6.3; Bio-Rad). Sample band density was divided by the average positive control sample band density (run two or three times in the same gel) to give relative band density.

### Calculations and statistics

Total protein concentrations were calculated by multiplying protein concentration in the initial homogenate by homogenate volume and dividing by tissue weight.

All data are presented as means ± SEM. Data were analyzed using JMP IN statistical software (Version 7.0.1; SAS Institute, Cary, NC, USA). P values of less than 0.05 were considered significant for all statistical tests. Tissue molybdenum data were analyzed using a Student’s 2 tailed t-test. Data from juvenile non-cannulated fish were submitted to a one-way ANOVA. Pre-exposure data from fingerling fish were submitted to a one-way ANOVA to test for exposure chamber effects while exposure data (8, 24, and 96 h) from fingerling fish were subjected to a two-way (main effects of treatment and sampling time) ANOVA. A Tukey-Kramer HSD test was applied to discern differences among means for those treatments that had statistically significant differences. Data from cannulated fish were analyzed by a repeated measures factorial design ANOVA with between subjects factors (characterizing variation within a fish and not the population). When necessary, post hoc contrasts were used to discern differences among means for those treatments that had statistically significant differences.

## Results

### Tissue Molybdenum

**Fig 1:**
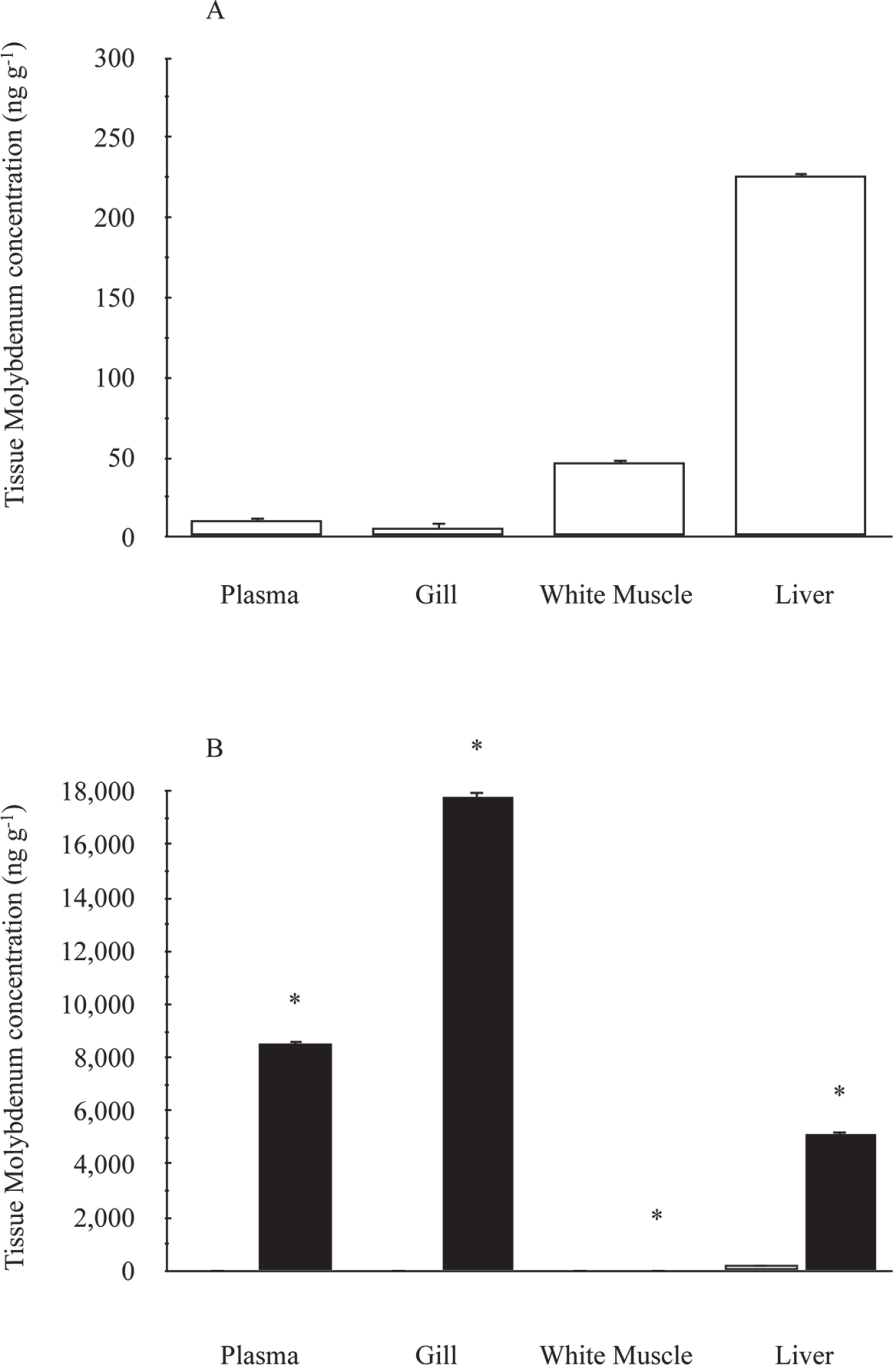
Tissue molybdenum concentration (ng g^-1^ tissue dry weight) in juvenile rainbow trout after 96 h exposure to waterborne Mo at a concentration of 20 mg l^-1^. Tissues from the control group (0 mg l^-1^ Mo) are displayed separately (A) and with (B) those from the Mo exposed group for clarity. Data plotted as means ± 1 SEM (n = 6). An asterisk indicates a significant difference with control using a paired, two-tailed Student’s t-test (α = 0.05)

Tissue molybdenum for gill, plasma, white muscle and liver of fish from the control group (0 mg l^-1^ Mo) to be 6.0 ± 3.46, 10.5 ± 2.02, 46.5 ± 1.44 and 227 ± 2.02 ng g^-1^ dry weight, respectively (Fig. 1A). Following 96 h exposure to 20 mg l^-1^ molybdenum, these tissues in juvenile rainbow trout were found to contain significantly greater molybdenum concentrations with gill and plasma showing the greatest increases (Fig. 1B). Gill and plasma molybdenum concentrations increased by 3,000 and 800 times, respectively, compared to the control group. Liver and white muscle experienced less dramatic, yet significant, increases in molybdenum concentration with a 23 fold and a 38% increases, respectively compared to the control group. The findings shown in Fig. 1 are consistent with those for tissue molybdenum concentrations from all juvenile fish exposed to elevated water molybdenum.

### Physiological stress response parameters

**Fig 2:**
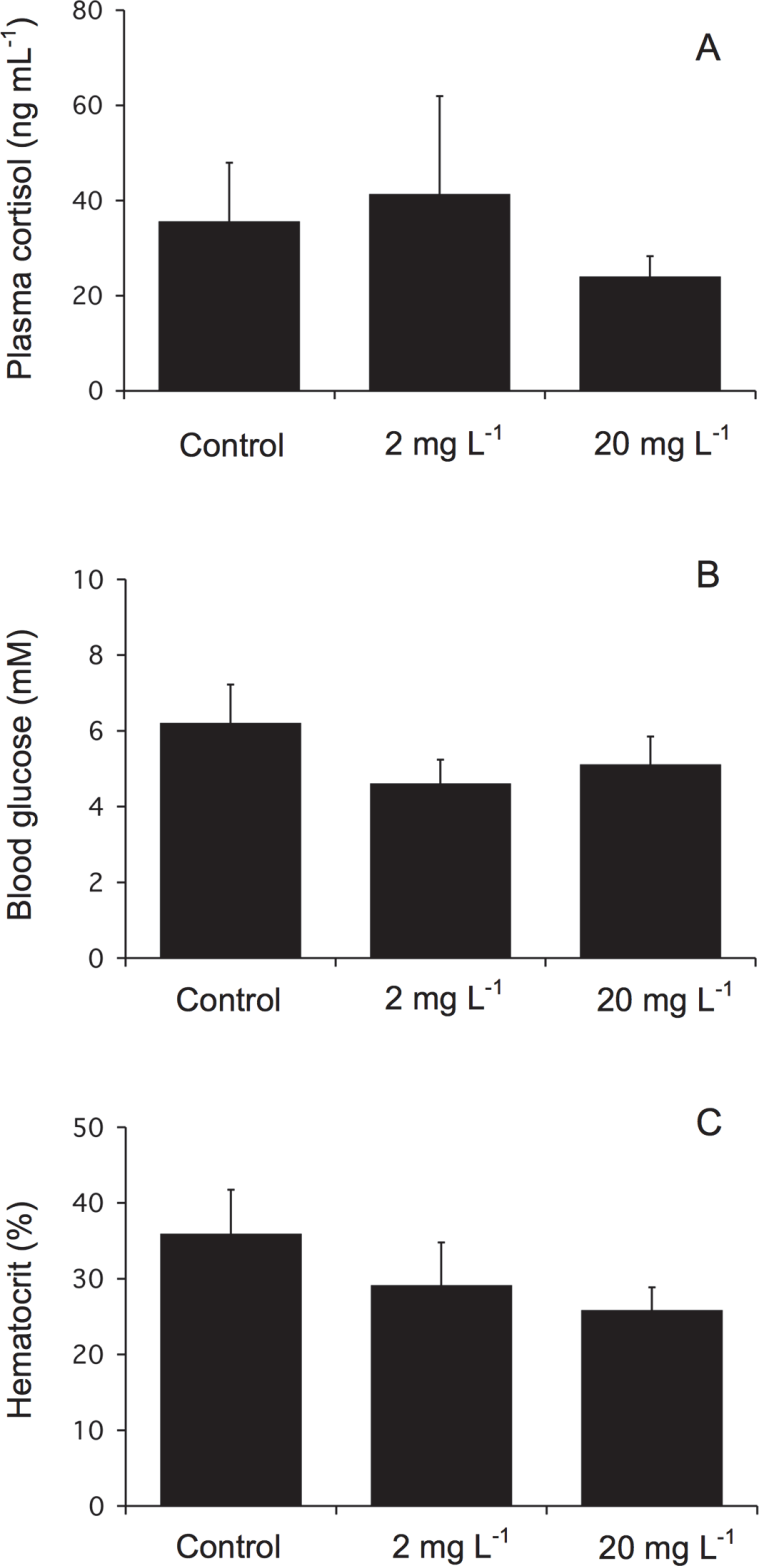
Plasma cortisol (A), blood glucose (B), and hematocrit (C) levels of juvenile rainbow trout exposed for 96 h to waterborne Mo at concentrations of 0, 2, or 20 mg l^-1^. Data plotted as means ± 1 SEM (n = 6). There were no significant differences (p > 0.05) between groups.

Plasma cortisol levels remained unchanged in all groups of juvenile fish after 96 h of exposure to 2 or 20 mg l^-1^ of Mo (Fig. 2A). There was also no detectable effect of Mo, at the same concentrations, on plasma cortisol at 8, 24, or 96 h in the cannulated juvenile fish (data not shown). The 96 h pooled treatment means in non-cannulated (33.6 ± 7.7 ng ml^-1^) and cannulated (26.1 ± 5.6 ng ml^-1^) fish were not significantly different.

In concordance with plasma cortisol data, Mo exposure to 2 or 20 mg l^-1^ did not have a significant effect on plasma glucose levels in juvenile fish when measured after 96 h (Fig. 2B). Similar results were observed during the time course (8, 24, and 96 h) studies in cannulated juvenile fish exposed up to 20 mg l^-1^ of Mo and fingerling fish exposed up to 1,000 mg l^-1^ (data not shown). However, in these two studies there was a decrease in glucose levels over time. No significant differences between the 96 h pooled treatment means in non-cannulated juvenile fish (5.3 ± 1.0 mM), cannulated juvenile fish (4.5 ± 1.0 mM), and fingerling fish (3.6 ± 0.2 mM) were detected.

At 96 h, hematocrit levels in juvenile fish exposed to 2 or 20 mg l^-1^ of Mo were not significantly different than control fish (Fig. 2C). Similar results were observed in the time course studies using cannulated juvenile fish and fingerling fish (data not shown). There was a significant difference in 96 h pooled treatment means between cannulated fish (16.0 ± 1.4%) and non-cannulated fish: juvenile fish (30.3 ± 2.9%) and fingerling fish (33.9 ± 1.9%).

### Cellular stress response parameters

**Fig 3:**
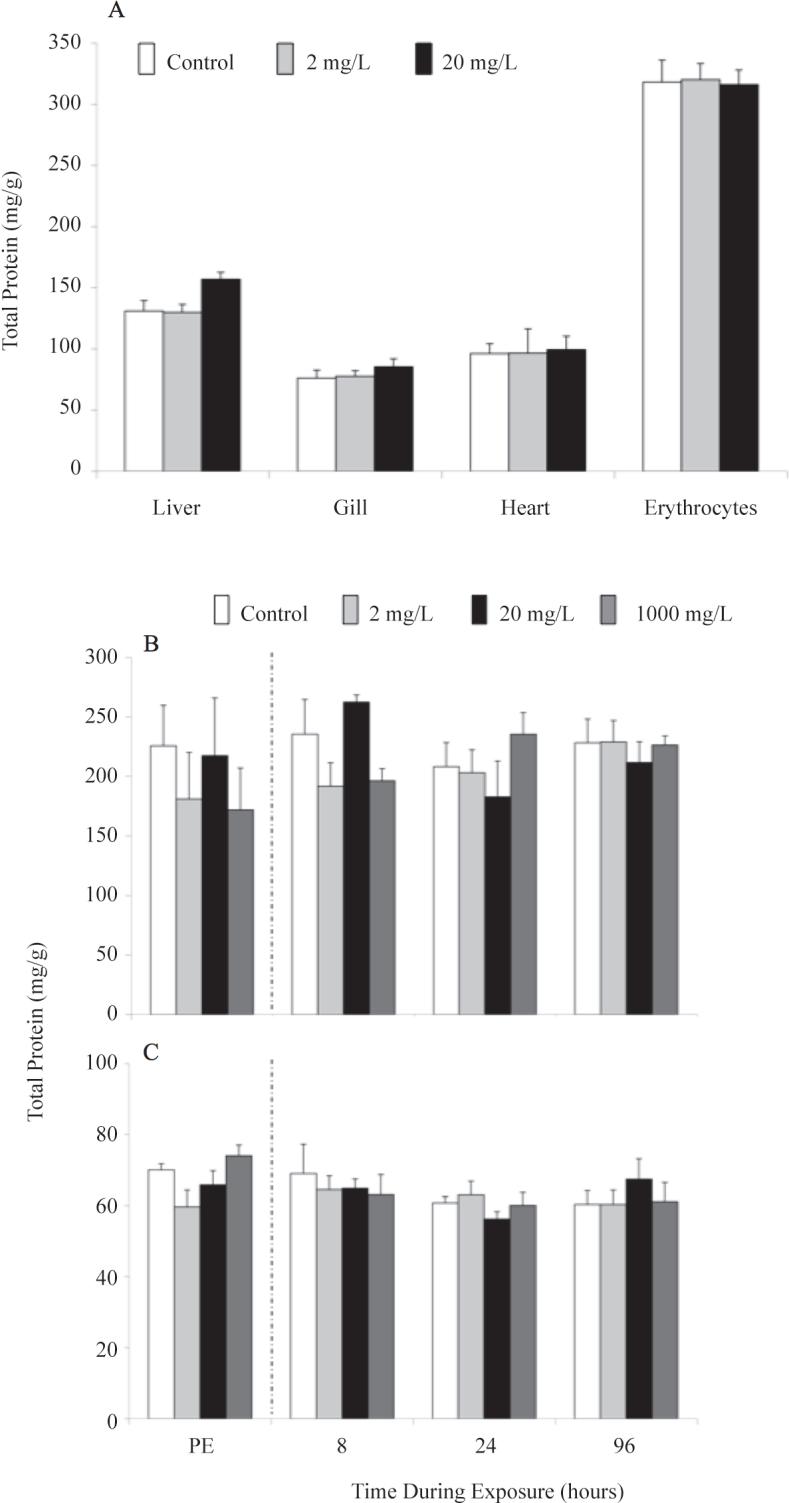
Tissue total protein concentration (mg g^-1^ tissue wet weight) in rainbow trout after 96 h molybdenum exposure. Tissue total protein is shown for liver, gill, heart and erythrocyte total protein in juvenile trout exposed to 0, 2 or 20 mg l^-1^ (A) and liver (B) and gill (C) in fingerling trout exposed to 0, 2, 20 or 1,000 mg l^-1^molybdenum. Fingerlings were sampled prior to (PE = pre-exposure) and at 8, 24, and 96 h of exposure. Data plotted as means ± 1 SEM (n = 6). No significant differences (p>0.05) were found.

Total liver, gill, heart, and erythrocyte protein concentrations from juvenile fish sampled after 96 h of exposure to 2 or 20 mg l^-1^ of Mo showed no statistically significant differences when compared to controls (Fig. 3A). Similar results were observed in fingerling fish (Fig. 3 B, C). There were no significant differences in liver or gill total protein concentration in fish sampled from each of the exposure chambers (0, 2, 20, and 1,000 mg l^-1^) prior to exposure. During the exposure period all fingerlings responded with a lack of change in total protein in both tissues.

**Fig 4:**
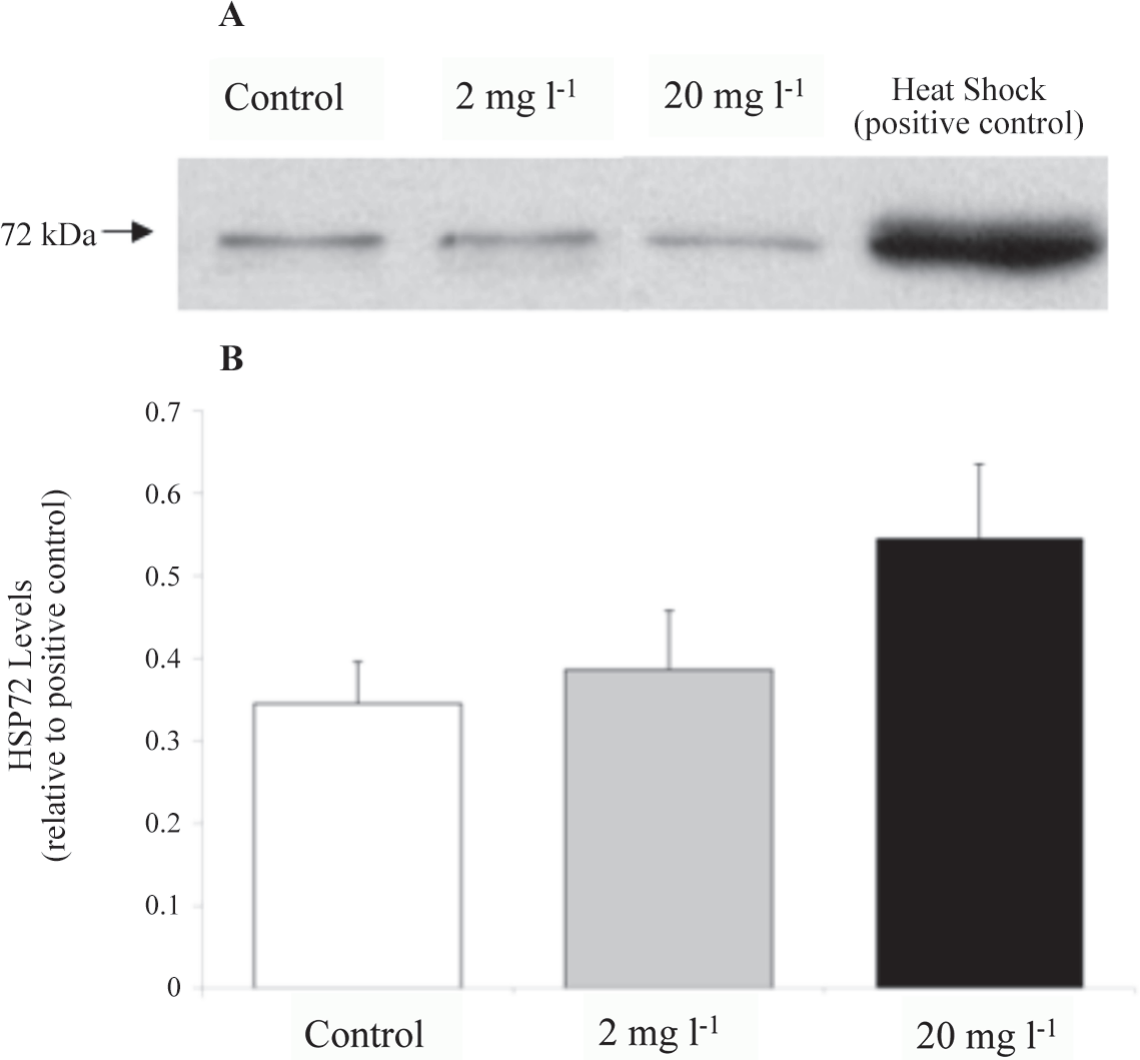
Erythrocyte hsp72 levels in from juvenile rainbow trout exposed to molybdenum (0, 2, or 20 mg l^-1^) for 96 h. (A) Representative Western blot of hsp72 expression in juvenile rainbow trout erythrocytes. (B) Erythrocyte hsp72 levels (relative to positive control). Data plotted means ± 1 SEM (n = 6). There were no significant differences (p > 0.05) between groups.

Immunoblotting was carried out with a panel of hsp antibodies on cell free extracts of liver, gill, heart, and erythrocyte samples from fish exposed to Mo. There was no detectable induction of hsp72 under control conditions or after 96 h of exposure to 2 or 20 mg l^-1^ in the liver, gills, or heart of juvenile fish. The erythrocytes, however, in both the control group and the Mo exposure groups expressed basal levels of hsp72 at about 30–50% of the heat shocked positive control (Fig. 4). Even at 1,000 mg l^-1^ there was no detectable induction of hsp72 in the liver or gills of fingerling fish.

**Fig 5:**
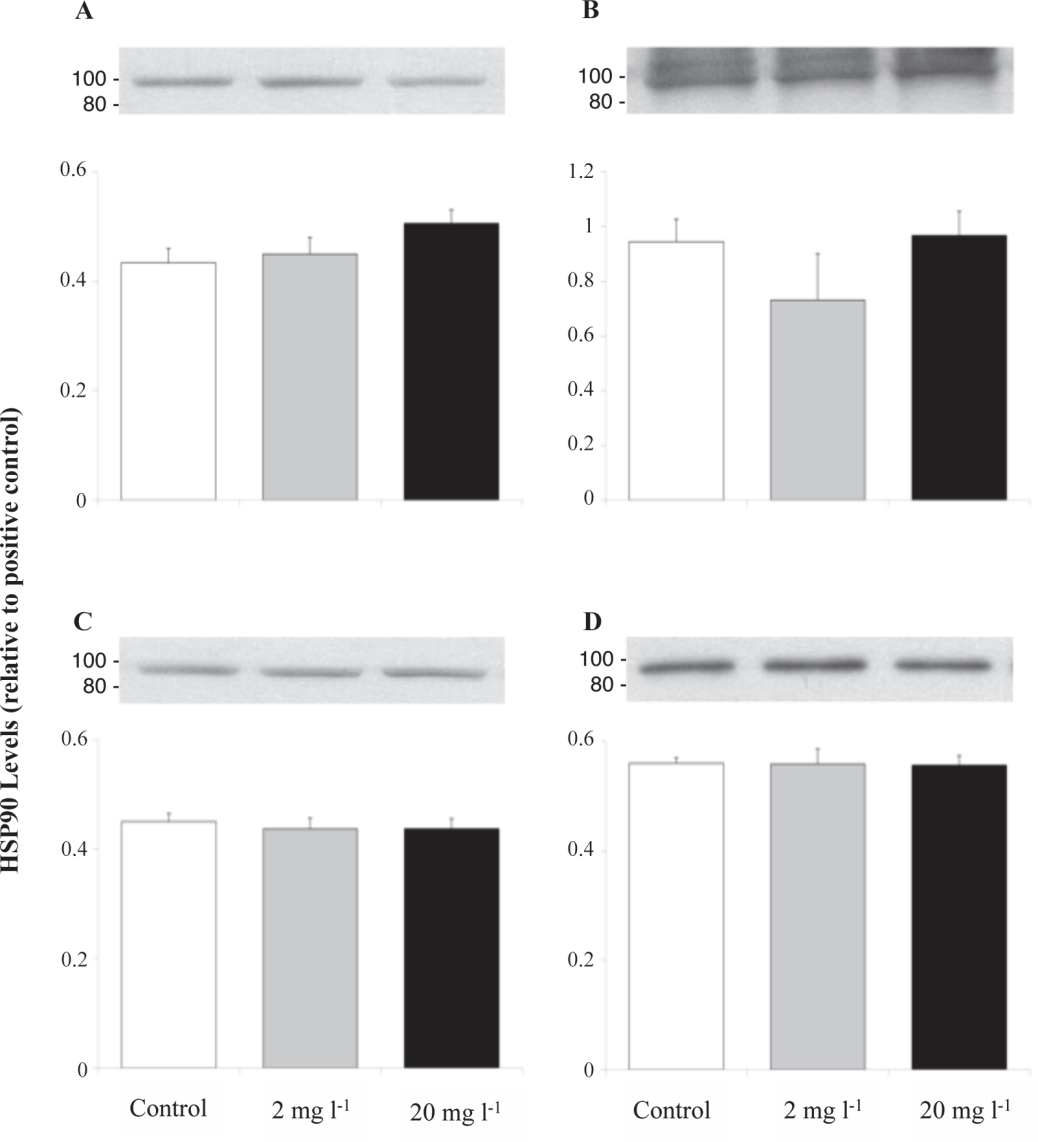
Liver (A), gill (B), heart (C), and erythrocyte (D) total hsp70 levels (relative to positive control) of juvenile rainbow trout exposed for 96 h to waterborne Mo at concentrations of 0, 2, or 20 mg l^-1^. Data plotted as means ± 1 SEM (n = 6). There were no significant differences (p > 0.05) between groups. Representative Western blots for total hsp70 in the each tissues are shown above their respective bar graphs with the location of location of the MagicMark XP Wester nProtein Standard indicated on the left side of each blot (in kDa).

**Fig 6:**
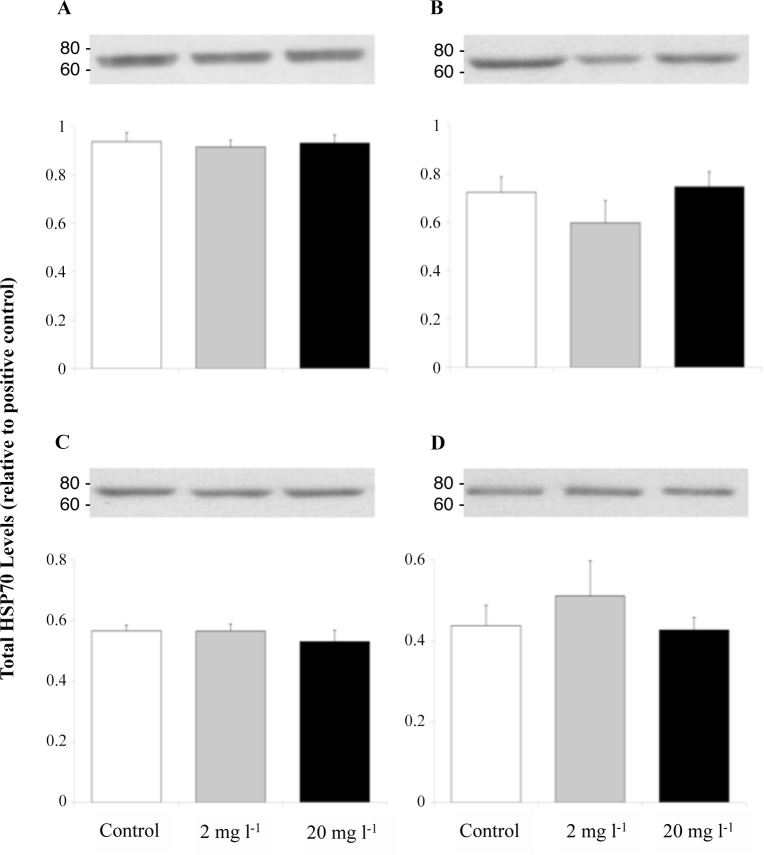
Liver (A), gill (B), heart (C), and erythrocyte (D) hsp90 levels (relative to positive control) of juvenile rainbow trout exposed for 96 h to waterborne Mo at concentrations of 0, 2, or 20 mg l^-1^. Data plotted as means ± 1 SEM (n = 6). There were no significant differences (p > 0.05) between groups. Representative Western blots for total hsp70 in the each tissues are shown above their respective bar graphs with the location of location of the MagicMark XP Western Protein Standard indicated on the left side of each blot (in kDa).

**Fig 7:**
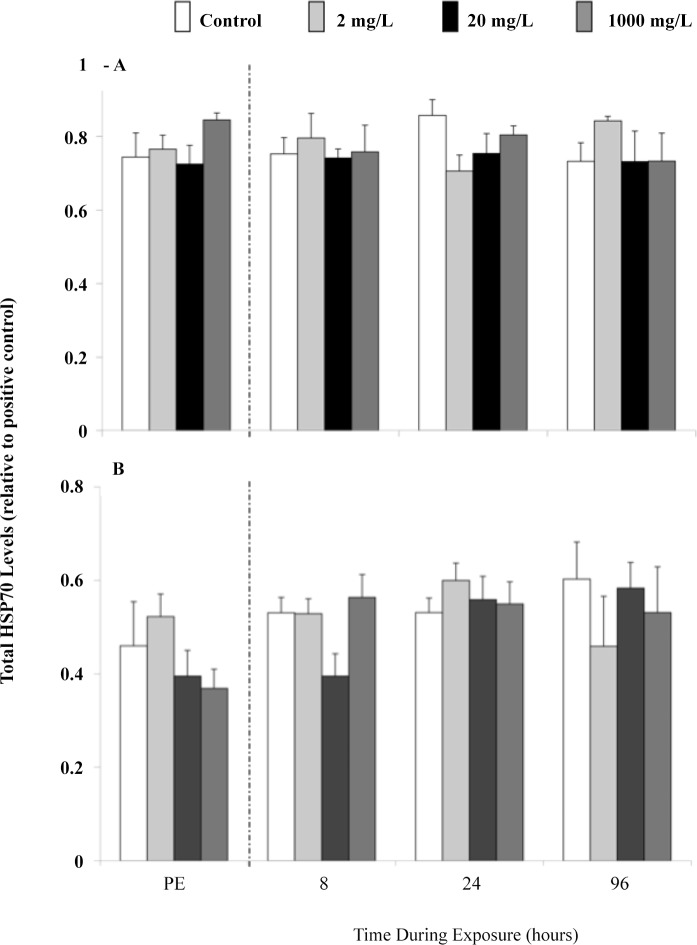
Liver (A) and gill (B) total hsp70 levels in fingerling rainbow trout after a 96 h molybdenum exposure to 0, 2, 20 or 1,000 mg l^-1^. Fish were sampled prior to exposure (PE = pre-exposure) and at 8, 24 and 96 h during exposure. Data plotted as means ± 1 SEM (n = 6). No significant differences (p > 0.05) were found.

**Fig 8:**
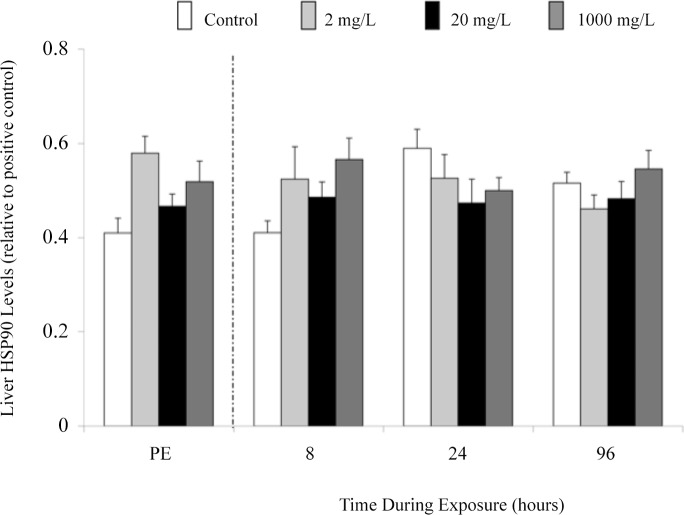
Liver hsp90 levels (relative to positive control) in fingerling rainbow trout exposed to 0, 20, or 1,000 mg l^-1^ of molybdenum prior to exposure (PE = pre-exposure) and during exposure (8, 24 and 96 h). Data presented as means ± 1 SEM (n = 6). No significant differences (p > 0.05) were found.

Figs. 5 and 6 show that the expression of total hsp70 (72 and 73) and hsp90 in the liver (A), gills (B), heart (C), and erythrocytes (D) of juvenile rainbow trout remained at control levels after the 96 h exposure to 2 or 20 mg l^-1^. Fingerling fish sampled prior to addition of Mo did not differ in their liver and gill total hsp70 (Fig. 7A, B) or liver hsp90 (Fig. 8) levels, and these levels did not change when sampled at 8, 24, and 96 h during exposure.

**Fig 9:**
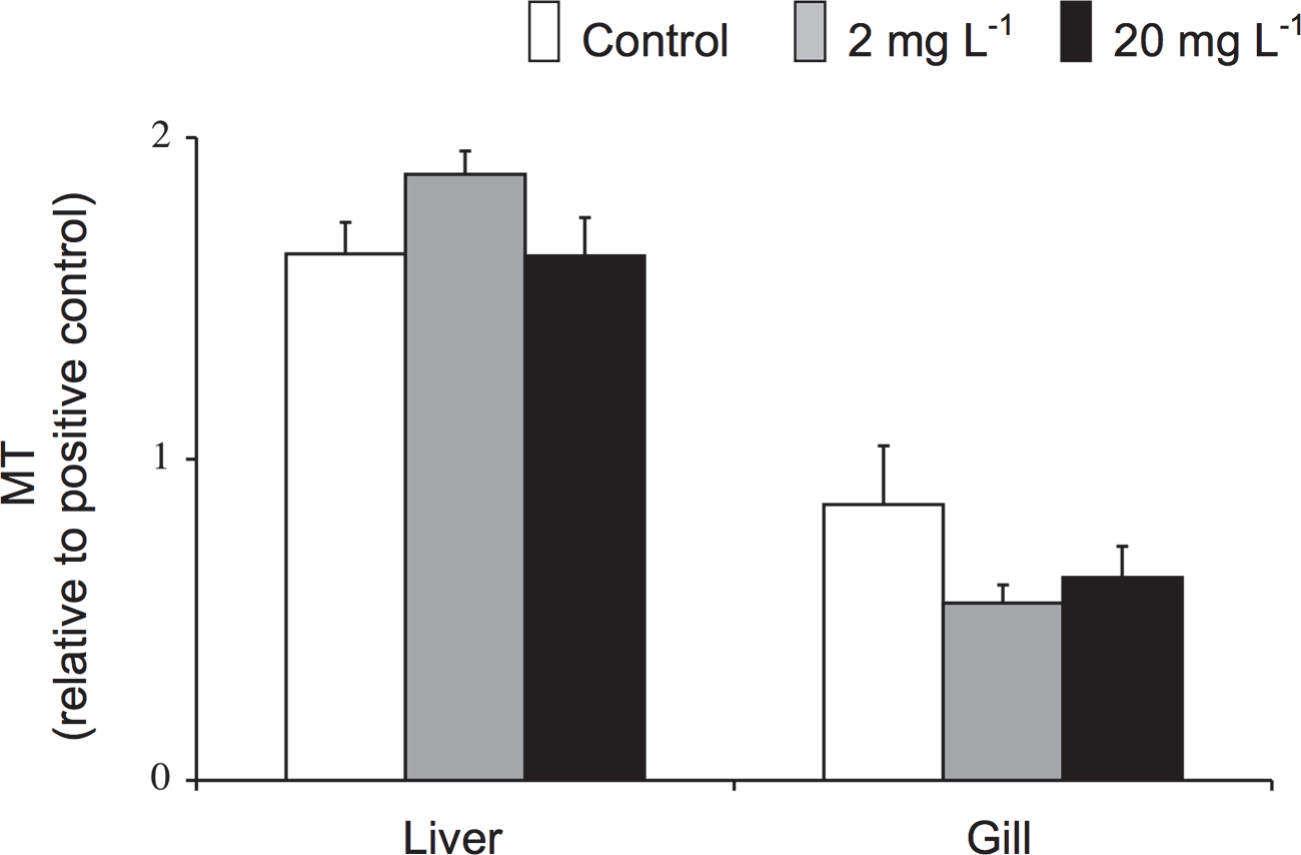
Liver and gill MT levels (relative to positive control) of juvenile rainbow trout exposed for 96 h to waterborne Mo at concentrations of 0, 2, or 20 mg l^-1^. Data were plotted as means ± 1 SEM (n = 6). There were no significant differences (p > 0.05) between groups.

**Fig 10:**
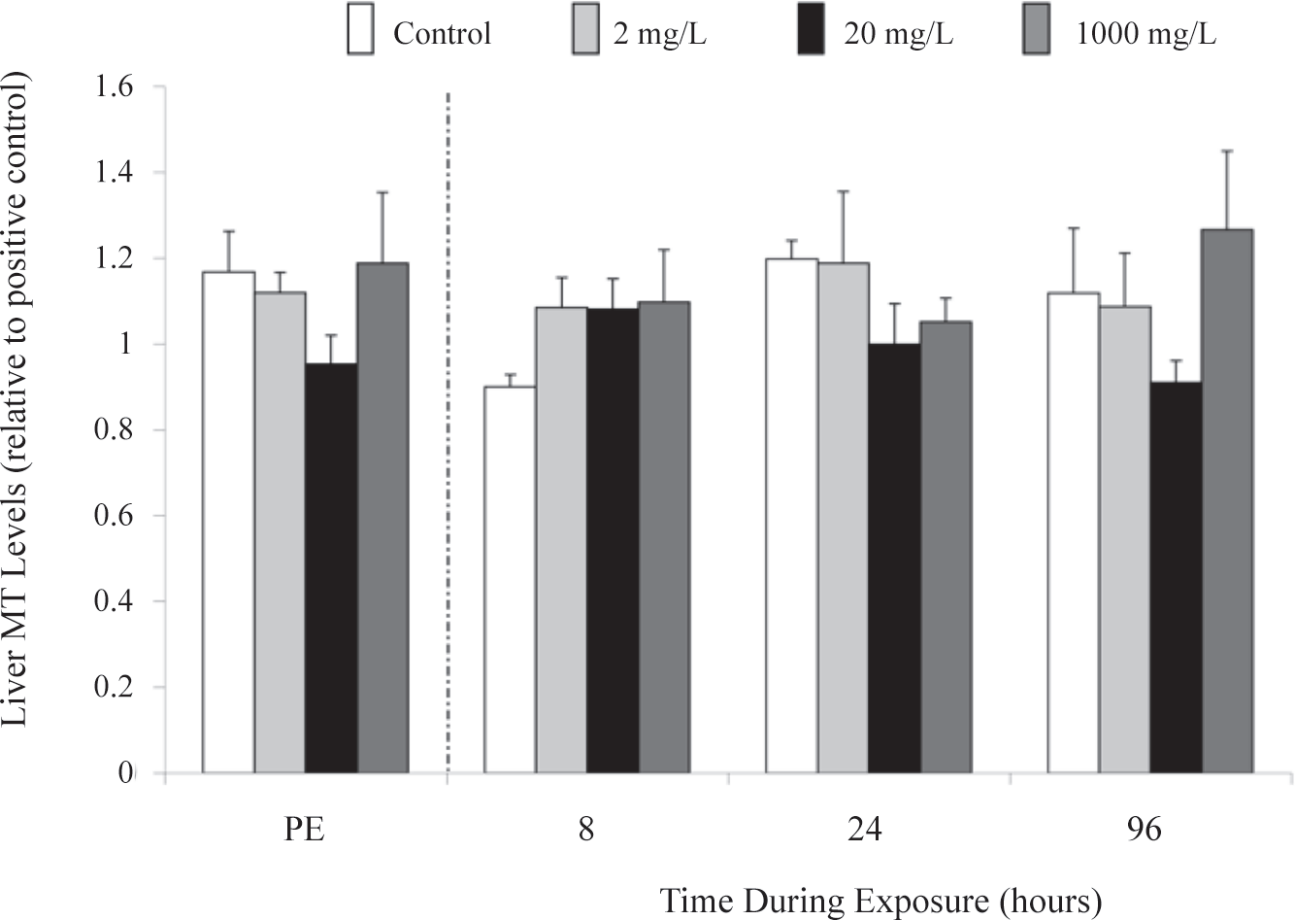
Liver MT levels (relative to positive control) in fingerling rainbow trout after a 96 h exposure to 0, 2, 20, or 1,000 mg l^-1^ of molybdenum. Fish were sampled prior to (PE = pre-exposure) and after 8, 24, and 96 h of exposure. Data plotted as means ± 1 SEM (n = 6). No significant differences (p > 0.05) were found.

Analysis of MT band density in liver and gill tissue of juvenile fish did not reveal any significant differences between control fish and Mo exposed (2 or 20 mg l^-1^) fish after 96 h of exposure (Fig. 9). Similar results were observed in fingerling fish exposed to up to 1,000 mg l^-1^ when liver (Fig. 10) and gill tissue were examined prior to exposure and at 8, 24, and 96 during exposure.

## Discussion

Fish in nature are exposed to a variety of stressors that can adversely affect their health. In order to cope with stress, fish can respond to it by eliciting a physiological or a cellular stress response. Such responses result in biochemical, hematological, and cellular changes that can be used as biomarkers to allow for the assessment and management of stress in fish. While there have been many studies correlating various stress related biomarkers with exposure to metals such as cadmium [57], copper [58], and zinc [59], information concerning Mo is extremely limited. This study investigated the effects of an acute, sublethal Mo exposure on the stress response of rainbow trout. Both fingerling and juvenile fish did not elicit a physiological or a cellular stress response when exposed to Mo nor were there any detectable differences in sensitivity between the two life stages despite the tissue accumulation of significant molybdenum (Fig. 1, this study; [26]).

Control plasma cortisol concentrations were within the range of values reported for unstressed rainbow trout [29, 32, 43, 60–63]. A Mo exposure of up to 20 mg l^-1^ did not activate a plasma cortisol response in juvenile rainbow trout at 8, 24, and 96 h during exposure. Results from this study are consistent with data from a 168 h endpoint Mo exposure to 25 or 250 mg l^-1^ in kokanee salmon reporting no difference in plasma cortisol levels between Mo exposed and control fish [26].

It has been suggested that the effects of toxicants on the stress response vary with the nature of the chemical, its concentration, method of exposure, and duration of exposure. For example, a waterborne exposure to the 96 h LC_50_ of copper (0.25 mg l^-1^) caused plasma cortisol levels to increase by ~280 ng ml^-1^ after 24 h of exposure [32]. Similarly, plasma cortisol levels increased ~140 ng ml^-1^ at 72 h during exposure to cadmium [0.01 mg l^-1^, 50% of the 96 h LC_50_ determined by Hollis et al. [64]; Chowdhury et al., 2004] and increased ~65 ng ml^-1^ after 96 h of exposure to selenite (2.52 mg l^-1^, 35% of the 96 h LC_50_ determined by [36, 65]). In contrast, but similar to the results of this study, waterborne exposure to lower doses of copper (0.016 mg l^-1^; 6.4% of the 96 h LC_50_ determined by Syvokiene et al. [66]) for 72 h [61] and selenite (0.72 mg l^-1^, 10% of the 96 h LC_50_ determined by Hodson et al. [65]) for 96 h [36] did not elevate plasma cortisol levels. All of the aforementioned exposure studies used rainbow trout as the test species. Therefore, it is likely that in this study 20 mg l^-1^, 0.27% of the 96 h LC_50_ reported by Bentley [21] whose study used rainbow trout of similar size to rainbow trout in this present study, was not concentrated enough to elicit a response.

Control glucose and hematocrit levels were within the range previously reported in unstressed rainbow trout [29, 35, 67–70]. The lower hematocrit values observed in the cannulated fish versus the non-cannulated fish are characteristic of cannulated fish [71–74]. Explanations may be blood loss due to the cannulation procedure and mild hemodilution, caused by repeated sampling and injection of saline after each sampling. In the present study, the absence of hyperglycemia is consistent with the lack of elevated cortisol. Although hyperglycemia was not observed throughout the 96 h exposure period, a hypoglycemia was observed. The decline in glucose is most likely a result of withholding feed during acclimation and experimentation and the exhaustion of energy stores. Brown et al. [75] reported a similar alteration in plasma glucose in catheterized rainbow trout and attributed it to withholding feed. There was no effect of waterborne Mo exposure on hematocrit. The results of this study are in concordance with data from a chronic (1 year) waterborne exposure of up to 17 mg l^-1^ reporting no change in hematocrit in various life stages of rainbow trout [73]. Findings outlined by McConnell [73] regarding observations of fused gill lamellae in rainbow trout and by Reid [26] regarding increased ventilation and mucus production in kokanee salmon during Mo exposure, however, would preclude one to think that these manifestations would have an effect on hematocrit. According to Heath [76], any pollutant that results in gill damage and subsequent internal hypoxia can be expected to increase hematocrit. This indicates that waterborne Mo, despite irritating the gills, does not induce internal hypoxia. This is also true of the metal lead. When Hodson et al. [77] and Martinez et al. [78] exposed rainbow trout and *Prochilodus lineatus* to waterborne lead they observed that although the metal caused changes in gill morphology hematocrit remained unaffected.

Exposure of rainbow trout to Mo failed to upregulate expression of hsp72, hsp73, and hsp90 (Figs. 4–8). There was no response in the liver, gills, heart, or erythrocytes of juveniles exposed to a maximum of 20 mg l^-1^ or in the liver or gills of fingerlings exposed to a maximum of 1000 mg l^-1^. As a result, there appears to be no utility of these proteins as measures of Mo exposure. There is confidence that the lack of induction in response to acute Mo exposure in trout does not reflect a reduced capacity of fish to activate a heat shock response. In this study, heat shocked fish responded by synthesizing hsp72 and in previous studies that used the same antibodies heat shocked fish responded with inductions in hsp72 and hsp90 in rainbow trout liver, heart, and erythrocytes [79, 80]. Heat shock in rainbow trout has also lead to increases in hsp70 mRNA in the liver, gills, heart, and blood [81]. The lack of hsp induction by Mo is also not due to metal load sequestering by MT because, as discussed later, there was no induction of MT in response to Mo exposure.

Molybdenum is not the only stressor that is incapable of stimulating hsp70 production. Neither anesthesia administration nor handling induced hsp70 levels in the liver, gills, heart, or muscle of rainbow trout [82]. Furthermore, various forms of husbandry stress such as anesthesia, hypoxia, capture, crowding, feed deprivation, and cold stress had no affect on hsp70 mRNA levels in the gills of Atlantic salmon [83]. The commonality of these stressors is that none of them have been demonstrated to denature proteins. Therefore, it can be assumed that Mo, at concentrations tested in this study, does not cause detectable proteotoxicity.

Although a number of metals induce MT synthesis, there is a general assumption that Mo does not have this ability. Jakobsen et al. [84] reported MT induction in the liver of rats implanted with cobalt-chromium-molybdenum alloys, yet the authors speculated the induction as a response to the presence of cobalt, chromium, manganese, iron, and/or nickel but not to Mo. Koizumi et al. [85] demonstrated that Mo did not elevate levels of MT mRNA; however, increases in mRNA are not always concomitant with increases in protein. This study is the first to suggest that Mo does not stimulate MT protein expression. Mo exposure of concentrations up to 1,000 mg l^-1^ did not cause an up-regulation of MT in the liver or gills of rainbow trout (Figs. 9, 10), tissues that are known to possess high levels of MT [86] and accumulate Mo (Fig. 1, this study; [26]). This finding suggests that MT levels cannot be used as an indicator of previous environmental exposure to Mo. The lack of MT induction suggests that Mo neither induces MT directly through binding to MT nor indirectly through activation of an inflammatory response.

Molybdenum is a borderline, d(5) metal [87]. As such, the metal has significant oxide and sulphide chemistries as demonstrated by its formation of molybdenite (MoS_2_) and wulfenite (PbMoO_4_). As a result, Mo can be expected to bind to the negatively charged thiolate groups of MT if it exists as a cation. Other borderline d(5) metals such as chromium and manganese can bind to MT but with low affinity [88]. Lead, for example, has a high affinity for MT *in vitro* [89] but binds sparingly *in vivo* [90]. The lack of MT induction, however, suggests that Mo did not bind to MT. These findings would be expected if Mo, which exists as molybdate in the natural environment and has been shown to move across the gill as molybdate (J. Hoeskstra and S.D. Reid, pers. comm.) was distributed internally as molybdate. Although the speciation of Mo in fish body fluids has yet to be characterized, the interpretation of our MT findings are consistent with the study by Matsuura et al. [91] that demonstrated that Mo exists as molybdate inside salmon egg cytoplasm.

Exposure to chromium, iron, cobalt, nickel, arsenic [92, 93], cerium [94], and vanadate [95], metals which are unable to bind to MT, caused inductions in MT but indirectly though an inflammatory mediated response. These metals inflicted tissue injury triggering the production of cytokines such as interleukin-1 beta, interleukin-6, and tumor necrosis factor alpha, all known inducers of MT [96]. The fact that Mo did not increase MT expression also suggests that this metal is unable to induce an inflammatory response. Therefore, the toxic response of fused gill lamellae reported by McConnell [22] is probably not true tissue damage but rather a response to ionoregulatory disruption.

The findings from this study are consistent with previous studies demonstrating that an elevation in Mo is not perceived as a toxic threat by fish [22, 23, 26]. Collectively, these studies and the current one bring into question the Mo water quality guidelines for the protection of freshwater aquatic life of 0.073, 0.04, and 2 mg l^-1^ set by Canada [2], Ontario [97], and British Columbia [55], respectively. It is therefore suggested that the current limits may be overly protective of rainbow trout and should be reevaluated. However, based on our limited finds we are not suggesting that the limit should be set to 1,000 mg l^-1^. This study involved the acute exposure of only two life stages of a single species of freshwater fish to Mo, the analysis of an incomplete suite of physiological indicators of stress/toxicity and we have previously observed mortality in juvenile freshwater fish (kokanee) exposed to 2,000 mg l^-1^ molybdenum under nearly identical conditions [26]. Furthermore, there is evidence that Mo in combination with additional environmental stressors could contribute to increased mortality in fish [23, 26, 98, 99]. Therefore a much or comprehensive examination of the toxicity of molybdenum is warranted and completed before any change in the Mo water quality guidelines are consider.
